# Electronic Cigarettes and Indoor Air Quality: A Simple Approach to Modeling Potential Bystander Exposures to Nicotine

**DOI:** 10.3390/ijerph120100282

**Published:** 2014-12-24

**Authors:** Stéphane Colard, Grant O’Connell, Thomas Verron, Xavier Cahours, John D. Pritchard

**Affiliations:** 1 Imperial Tobacco Limited, Winterstoke Road, Bristol BS3 2LL, UK; E-Mails: grant.oconnell@uk.imptob.com (G.O.C.); john.pritchard@uk.imptob.com (J.D.P.); 2 SEITA, Imperial Tobacco Group, 48 rue Danton, 45404 Fleury-les-Aubrais, France; E-Mails: thomas.verron@fr.imptob.com (T.V.); xavier.cahours@fr.imptob.com (X.C.)

**Keywords:** air quality modelling, indoor air pollution, e-cigarette, bystander exposure, nicotine

## Abstract

There has been rapid growth in the use of electronic cigarettes (“vaping”) in Europe, North America and elsewhere. With such increased prevalence, there is currently a debate on whether the aerosol exhaled following the use of e-cigarettes has implications for the quality of air breathed by bystanders. Conducting chemical analysis of the indoor environment can be costly and resource intensive, limiting the number of studies which can be conducted. However, this can be modelled reasonably accurately based on empirical emissions data and using some basic assumptions. Here, we present a simplified model, based on physical principles, which considers aerosol propagation, dilution and extraction to determine the potential contribution of a single puff from an e-cigarette to indoor air. From this, it was then possible to simulate the cumulative effect of vaping over time. The model was applied to a virtual, but plausible, scenario considering an e-cigarette user and a non-user working in the same office space. The model was also used to reproduce published experimental studies and showed good agreement with the published values of indoor air nicotine concentration. With some additional refinements, such an approach may be a cost-effective and rapid way of assessing the potential exposure of bystanders to exhaled e-cigarette aerosol constituents.

## 1. Introduction

There has been rapid growth in the use of electronic cigarettes (e-cigarettes) in Europe, North America and elsewhere. For example, a report published in July 2014 by Action on Smoking and Health estimated as many as 2.1 million adults in the UK currently use electronic cigarettes [1]. With such increased prevalence, there is currently a debate on whether the aerosol exhaled following the use of e-cigarettes has implications for the quality of air breathed by bystanders. E-cigarettes are small battery powered devices, commonly resembling cigarettes with a built-in or replaceable reservoir for an “e-liquid”, consisting primarily of propylene glycol, glycerol, nicotine and flavourings. When the user takes a puff on the product, a heating element is activated converting the liquid into an aerosol, which is then taken into the mouth or inhaled, and subsequently exhaled. A number of studies have reported that nicotine, amongst other chemical compounds, is released into the air during use of e-cigarettes [2,3,4]. A review of the current scientific literature indicates that there is insufficient evidence from which to assess the impact that exhaled vapour has on indoor air quality summarised by [5,6]. Despite this, WHO has recommended banning their use in public places [7]. At the same time, there have been calls by some in the public health community to further understand such products both in terms of potential benefits and risks before establishing controls [8]. It is in this context that the assessment of a bystander’s exposure may be useful as one facet of the development of evidence based regulations.

Assessing indoor air quality through chemical analysis can be complicated and requires specialist techniques for sampling and analysis as documented in the ISO 16000 series of international standards of measurements of indoor air quality parameters e.g., [9,10]. An individual experiment or study of a single indoor space or environment may be expensive and time intensive to conduct and may not be widely generalizable to other settings. However, as with many other questions of air quality, a range of mathematical tools have been developed by regulatory bodies and academic centres to explore air quality effects from a diverse range of chemical sources [11,12]. Such tools have been developed, tested and validated as predictive models of both outdoor and indoor environments [13,14]. The inherent flexibility in such modelling allows the rapid evaluation of different indoor spaces and the factors influencing exposure to be understood in detail.

A standard approach for modelling a microenvironment system, for example an office, is to employ mass balance equations. This is based on the general physical law of conservation of mass, to calculate the concentrations in indoor settings from knowledge of the source strength, the volume of the indoor environment, the effective air exchange rate, and the pollutant loss rate due to deposition and/or chemical reactions. This law accounts for all the mass emitted, present, or lost, and allows the prediction of pollutant concentrations. Mass balance models have been developed, tested and validated to accurately predict the concentrations of substances present in indoor settings due to cigarette smoking activity [15].

In this paper, we outline the development of a simple air quality model that describes the concentration of e-cigarette aerosol constituents, including nicotine, in the ambient air in a typical, small office space. Additionally, influencing factors such as proximity and time in a room following use of an e-cigarette were incorporated to predict bystander exposure. The effect of the influencing factors were assessed and the model predictions were tested by comparing the resulting solution with published experimental data; its utility is illustrated by presenting a simulated scenario and the areas are identified which still need further experimental investigations.

## 2. Experimental Section

### 2.1. Model Inputs

The model developed here is used to predict indoor air concentrations of chemical constituents based on the successive steps that occur between inhalation of the aerosol by the e-cigarette user and the air breathed in by the bystander (termed “puff phases”) and the characteristics of the indoor environment. Puff phases and the model input parameters are reported in Table 1.

**Table 1:** Model inputs.

Description	Phases	Parameters
Inhalation/Exhalation	Inhalation of an aerosol following e-cigarette puff Retention of chemical retained by the body Exhalation of aerosol into the ambient indoor air	Quantity of aerosol chemical constituent inhaled/exhaled (µg) Retention rate of chemical (%) Puff frequency Number of puffs in each vaping session
Aerosol propagation/dilution	Propagation of the exhaled aerosol in the surrounding space Dilution of the exhaled aerosol in the ambient air	Speed of aerosol propagation in x,y,z directions (m/min) Volume of the indoor environment (m^3^)
Bystander exposure	Peak exposure Exhaled aerosol dilution in ambient air continues	Bystander distance from e-cigarette user (m)
Air exchange	Renewal of indoor air Surface deposition/desorption	Air exchange rate (per hour) Surface deposition/desorption velocity (m/min)
Bystander exposure dose	Breathing during a certain period of time	Breathing pattern (L/min) Time spent in the indoor environment (min)

### 2.2. Mathematical Formulation

Typically, indoor air quality models assume a well-mixed room with single constant emission source [16]. In that case, each point in a room is assumed to have the same instantaneous “pollutant” concentration as all other points in the room following release of the pollutant. This assumption is not entirely accurate for an intermittent source, in this case use of an e-cigarette, as potential bystander exposure to chemicals in the exhaled aerosol will be underestimated [17]. If one assumes immediate uniform mixing, the peak exposure at the bystander’s position is not considered at the early stages of exhaled aerosol propagation. The following sections describe how the sequential phases can be mathematically formalised in a simple way. A more detailed description is available as supplementary information (Supplementary Files)

#### 2.2.1. E-Cigarette User Inhalation and Exhalation

The quantity of the chemical constituent exhaled into the indoor environment is the quantity of that chemical inhaled minus the fraction of that chemical retained by the consumer. Inhaled and exhaled quantities are simply linked by a rate of retention. The concentration of the chemical constituent in the exhaled air is the ratio of the mass to the volume of air exhaled.

#### 2.2.2. Aerosol Propagation

For simplicity, the initial shape of the exhaled aerosol was assumed to fill a cube having the volume equivalent to that of the exhalation. This simplification is justified by the fact that no given shape perfectly represents the actual shape, which in real-life would vary with each puff, and because a volume propagating from a cubic shape is far simpler to calculate than any alternative. Furthermore, regarding short term exposure, the main key factor is the speed at which the aerosol will propagate rather than the specific initial shape which will change rapidly over time. Each dimension of the initial cube increases at given speeds due to diffusion and convection transforming this shape into a cuboid. It is considered that the aerosol will fill homogeneously the volume which is itself expanding progressively. This approach corresponds to a low-scale well-mixed assumption for model dispersion and dilution over time in space, without resorting to complex computational fluid dynamic modelling [16].

#### 2.2.3. Change of the Constituent Concentration over Time

The change in concentration of the aerosol chemical constituents during the propagation phase is calculated from the ratio of the quantity of the constituent exhaled to the volume that is progressively filled. Once the exhaled constituent has filled a certain proportion of the room, extraction begins to be a factor from indoor ventilation mechanisms and/or leakage. The airflow extracted is derived from the air exchange rate and the volume of the room. If the air extracted is partly recycled, a recycling factor shall be included in the calculation. The absolute airflow of extraction is given by Equation (1):

(1) $$Q_{Extract}=(ACH/\text{6}0)\times V_{Room}\times \left(\text{1}00\;-RRA\right)$$

where

*Q_Extract_* = the extraction airflow (m^3^/min),
*ACH* = the air exchange rate (/hr),
*V_Room_* = the volume of the room (m^3^), and
*RRA* = the recycling rate of extracted air (%).

Extraction is not the only factor reducing the quantity of aerosol constituent in air; surface deposition may also contribute to reduction. The deposition velocity is the net flux density of a constituent to a surface divided by the concentration in the air. This velocity depends on numerous factors like aerosol particle size, surface natures or environmental conditions [18,19]. It is important to note that potential coagulation phenomena of aerosols and droplet evaporation may influence particle size changes as well as mass changes which may affect deposition velocity. Here, the equivalent airflow of deposition is a combination of adsorption/desorption phenomenon obtained from the multiplication of the surface by the velocity as expressed by Equation (2):

(2) $$Q_{Dep}=S_{Dep}\times v_{d}$$

where

*Q_Dep_* = the net deposition flow out of air to surface (m^3^/min),
*S_Dep_* = the surface of deposition (m^2^), and
*v_d_* = the net deposition velocity (m/min).

when both air extraction and surface deposition occur, the resulting reduction in the quantity of aerosol constituent in the ambient air can be expressed with the differential Equation (3):

(3) $$\frac{dM_{Constitutent}(t)}{dt}=-Q_{Extract}\times C_{Constituent}(t)-Q_{Dep}\times C_{Constituent}(t)$$

where

$M_{Constitutent}(t)$ = the quantity of aerosol constituent in the volume of the room (µg), and
$C_{Constituent}(t)$ = the concentration of aerosol constituent in volume filled (µg/m^3^).

Equation (3) represents simply the fact that the quantity removed from the air is either extracted and/or deposited. The volume of the room being constant, the change over time of aerosol constituent quantity is proportional to the change of the concentration. The differential Equation (4) can then be derived:

(4) $$\begin{array}{l} V_{Room}\times \frac{dC_{Constituent}\left(t\right)}{dt}=-\left(Q_{Extract}+Q_{Dep}\right)\times C_{Constituent}\left(t\right) \end{array}$$

The solution of Equation (4) is given by Equation (5):

(5) $$C_{Constituent}\left(t\right)=C_{Constituent\_Init}\times e^{-\left[\frac{Q_{Extract}+Q_{Dep}}{V_{Room}}\times (t-t_{Init})\right]}$$

where $C_{Constituent\_Init}$ = the constituent concentration when extraction and/or deposition starts (µg/m^3^), *t_Init_* = the initial time at which the extraction/deposition effects start (min).

If air extraction starts before the aerosol constituent completely fills the room, simultaneous effects of dilution and extraction during a certain period of time can be merged into a single expression as given with Equation (6):

(6) $$C_{Constituent}\left(t\right)=C_{Constituent\_Init}\times \frac{V_{Aerosol}\left(t_{Init}\right)}{V_{Aerosol}(t)}\times e^{-\left[a\times (t-t_{Init})\right]}$$

where $a=\frac{Q_{Extract}+Q_{Dep}}{V_{Room}}$

In reality, the effects of extraction and deposition may not occur simultaneously. A certain quantity of the constituent will be extracted by the ventilation system only once it has filled a sufficient proportion of the volume of the room, likewise for deposition. The coefficients “*a*” of Equation (6) can then change over time including events like a door or window opening.

#### 2.2.4. Exposure

Exposure is defined as the sum of the constituent concentration multiplied by the time a person is exposed [12]. At the position of the e-cigarette user, the concentration following exhalation is first influenced by dilution, and then by surface deposition and air exchange (Equation (6)). Once the aerosol constituent has reached the bystander, the peak exposure for the single puff is observed. Thereafter, the concentration around the bystander is influenced by dilution and by deposition and extraction.

#### 2.2.5. Total Quantity of Aerosol Constituent Inhaled by E-Cigarette User and Bystander

The total quantity inhaled by the e-cigarette user is given by Equation (7). This is the quantity of constituent inhaled during each puff and the quantity inhaled over time between puffs:

(7) $$\begin{array}{l} M_{Tot\_Inhaled\_User}=\sum_{1}^{N_{Puff}}M_{Constituent\_Inhaled}+\int_{0}^{t}C_{Constituent\_User}(t)\times V_{Inhaled}\cdot dt \end{array}$$

The total quantity inhaled by the bystander is given by Equation (8). This quantity is dependent on the fraction of the constituent retained or not by the e-cigarette user:

(8) $$M_{Tot\_Inhaled\_Bys\tan der}=\int_{0}^{t}C_{Constituent\_Bys\tan der}(t)\times V_{Inhaled}\cdot dt$$

(note: it was assumed that the constituent exhalation/re-emission by the bystander on concentration in air was considered negligible compared with the emissions from the e-cigarette user).

#### 2.2.6. Application to Multiple-Emissions, Multiple-Users and Multiple-Bystanders

Since the volume of the space is constant, the quantity of constituents exhaled and then the concentrations achieved by each successive puff and emission (single or multiple puffs) can be cumulated over time taking into account spatial positions. While the model developed here is centered on a single room, multi-room spaces could be considered in future work by coupling the differential equations for each zone and solving the equations numerically. However, the model would become more complex and specific to a given building architecture.

## 3. Results and Discussion

### 3.1. Model Output: “Single Puff Profile” and Exposure to Aerosol Constituents

**Figure 1:**
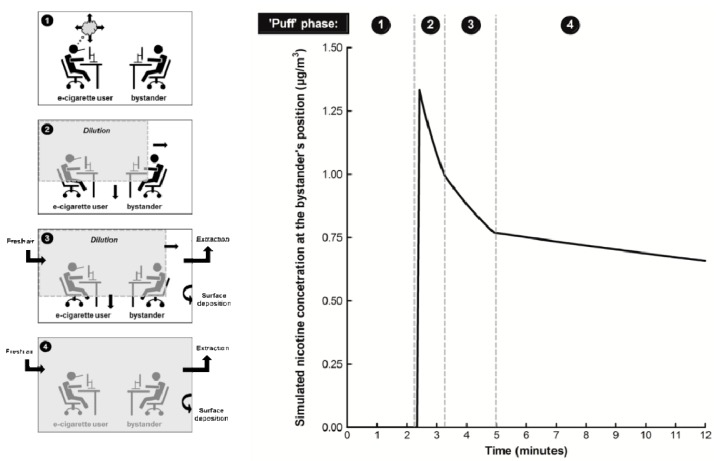
Phases of bystander exposure to exhaled vapour after a single “puff”. Phase **1**, the e-cigarette user takes a single “puff” and inhales the nicotine-containing vapour once every 10 min; the e-cigarette user exhales vapour into the indoor air and it propagates in all directions; the bystander not yet exposed to nicotine. Phase **2**, the bystander’s peak exposure to exhaled nicotine in the air is observed corresponding to the time the exhaled vapour reaches the position of the bystander; propagation of the exhaled vapour in the indoor air is not yet complete. Phase **3**, there is a reduction in the concentration of nicotine at the position of the bystander due to exhaled vapour propagation, dilution in the air and any surface deposition; it is assumed the air extraction starts when 80% of the room volume is filled with the exhaled vapour. Phase **4**, mixing of the exhaled vapour in the ambient air is complete and the concentration of nicotine is reduced by the air extraction and any surface deposition; there is a further reduction in the concentration of nicotine at the bystander’s position. Model inputs: distance from e-cigarette user, 2 m; quantity of nicotine exhaled, 30 µg per puff; speed of exhaled aerosol propagation in all directions, 0.6 m/min; air exchanges per hour, 1.33; and exhaled aerosol deposition velocity 0.06 m/min.

To assess potential exposure to a single exhaled aerosol constituent within the ambient air, e.g., nicotine, a number of input parameters and “puff phases” during e-cigarette use in the model are considered (Table 1). Following exhalation of a single “puff”, a profile of nicotine concentration in ambient air at the bystander’s position can be derived from the model (Figure 1). 

Following exhalation of the aerosol into the air, the aerosol propagates in all directions but initially the bystander is not exposed (phase 1). The peak exposure of the bystander to the constituent in the air corresponds to the time the propagated aerosol reaches the bystander’s position (phase 2). The concentration of the constituent in the indoor air then decreases at the bystander’s position due to the continued dilution and propagation of the aerosol in the room (phase 3) and the air extraction effects coupled with any surface deposition (phase 4).

### 3.2. Parameters Influencing Bystander’s Exposure

**Figure 2:**
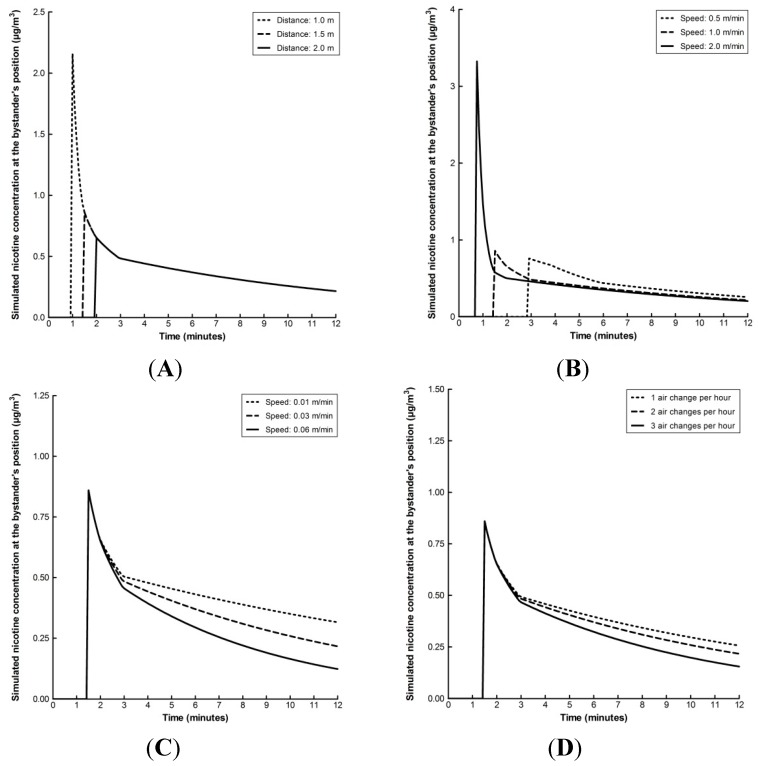
The effect of varying the value of one model input parameter one-at-a-time whilst all others remain constant. The effect of changing the distance of the bystander from the e-cigarette user from 1, 1.5 and 2 m (**A**); speed of exhaled aerosol propagation from 0.5, 1.0 and 2.0 m/min (**B**); exhaled aerosol speed of deposition from 0.01, 0.03 and 0.06 m/min (**C**); and air exchange rate from 1, 2 and 3 air changes per hour (**D**) on the simulated concentration of nicotine in the ambient air at the bystander’s position. When the model parameter value was not varied, it was fixed at the median value. In each case: distance from e-cigarette user, 1.5 m; quantity of nicotine exhaled, 20 µg per puff; speed of exhaled aerosol propagation, 1.0 m/min; exhaled aerosol deposition velocity 0.03 m/min; and air exchanges per hour, 2.

The model considers a number of input parameters as reported in Table 1. Here, the effect of changing the input value of these parameters in turn on the concentration of exhaled nicotine in the ambient air at the bystander’s position following a single “puff” is considered (Figure 2). The concentration of nicotine in the ambient air at the position of the bystander is reduced when the distance between the bystander and the e-cigarette user is increased e.g., from 1 to 2 m (Figure 2A). As the distance from the e-cigarette user is increased, the nicotine concentration breathed in by the bystander is reduced due to increased dilution of the exhaled aerosol in an increased volume of ambient air. When the speed of propagation of the exhaled aerosol in the direction of the bystander is increased from 0.5 to 2.0 m/min, all other speeds being kept constant, the time taken to reach the bystander is decreased and the peak of nicotine in the air at the bystander’s position is increased (Figure 2B). 

Once exposed, the faster the speed of dilution of the exhaled aerosol in the ambient air, the greater the decrease in the concentration of nicotine at the bystander’s position. By calculating the integrals of the curves over a one hour period, the changing speed of exhaled aerosol propagation has little effect on the bystander’s exposure: 10.3 µg/m^3^/min with a speed of aerosol propagation at 0.5 m/min; 10.8 µg/m^3^/min at 1 m/min and 13.3 µg/m^3^/min at 2 m/min. Next, the effect of the exhaled aerosol deposition velocity on the concentration of nicotine at the bystander’s position is considered (Figure 2C). As the deposition velocity is increased from 0.01 to 0.06 m/min, the concentration of nicotine at the bystander’s position is decreased and thus the bystander’s exposure is reduced from 17.8 to 6.1 µg/m^3^/min over a one hour period, respectively. Importantly, this does not follow a simple relationship of proportionality. Similarly, when the number of air changes per hour is increased, the concentration of nicotine in the ambient air at the bystanders position is reduced more rapidly (Figure 2D). Finally, the model indicates that the concentration of nicotine at the bystander’s position is directly proportional to the quantity of nicotine exhaled (data not shown). For example, when the quantity of nicotine exhaled is increased 3-fold, from 10 to 30 µg, the bystander’s exposure is also increased 3-fold, from 3.4 to 10.2 µg/m^3^/min. In summary, aerosol concentration at the bystander position is decreased with increased distance from the source, greater dilution, an increase in deposition speed and with increased air changes per hour.

### 3.3. Application of the Model

Using a range of parameters published in the scientific literature including e-cigarette nicotine delivery, emission pattern and room ventilation rate, the model can be used to predict the concentration of nicotine in indoor ambient air. This allows a comparison between modelled and experimental findings. In a recent study [2], a smoking machine was placed in a 39 m^3^ room and used to generate aerosol from three different brands of e-cigarettes. The concentration of nicotine delivered by each brand of e-cigarette (EC) was previously determined: EC1, 31 µg per puff; EC2, 58 µg per puff; EC3, 56 µg per puff [20]. For each e-cigarette brand, the machine generated aerosol was released into the room under four different experimental conditions: two intensities of aerosol emission pattern repeated after a 30 min interval corresponding to 7 puffs (“low” emission pattern) or 14 puffs (“high” emission pattern) every 10 s with “restricted” room ventilation (1.37 air changes per hour) or “intensive” room ventilation (12.6 air changes per hour). Two fans were used to mix the indoor air and an air sampling station was located 1 m from the smoking machine and 10 cm above the level of the e-cigarette product.

**Figure 3:**
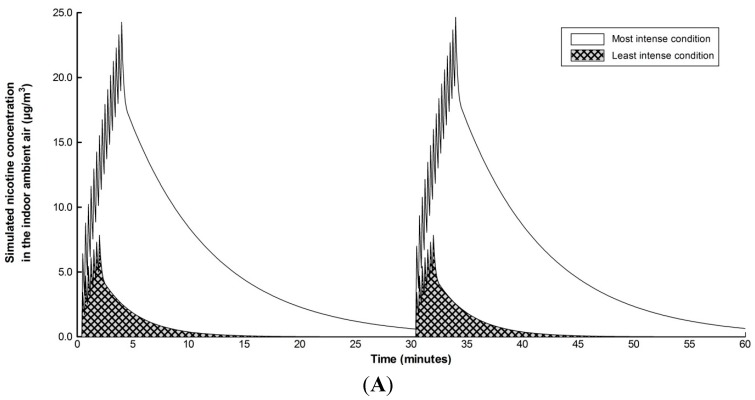

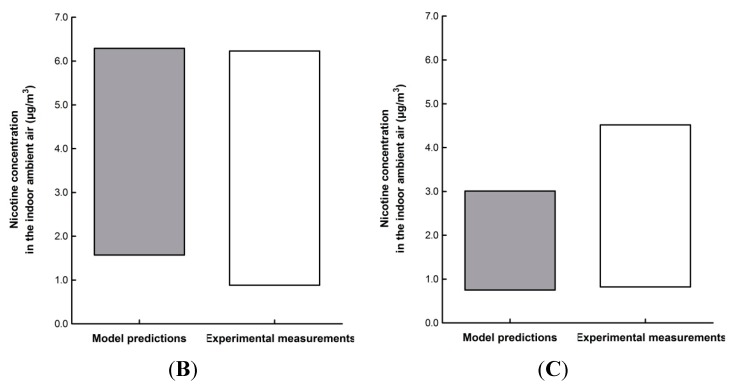
Estimation of the nicotine concentration in the indoor ambient air using experimentally-derived model input parameters. (**A**) Simulated change in nicotine concentration in the ambient air of a 39 m^3^ room over 1 h for two extreme experimental conditions. E-cigarette aerosols were generated using a smoking machine and released into the room under two extreme experimental conditions. The most intense condition corresponds to high e-cigarette nicotine delivery, 58 µg of nicotine delivered per puff; high aerosol emission pattern, 15 puffs every 10 s repeated at 30 min interval; and restricted room ventilation, 1.37 air changes per hour. The least intense condition corresponds to low e-cigarette nicotine delivery, 31 µg of nicotine delivered per puff; low aerosol emission pattern, 7 puffs every 10 s repeated at 30 min interval; and intensive room ventilation, 12.6 air changes per hour. To simulate the two extreme experimental conditions, the following assumptions were made: speed of aerosol propagation, 2 m/min; speed of aerosol deposition, 0.06 m/min; and nicotine retention, 0% (aerosols were generated using a smoking machine). (**B**,**C**) Comparison between model predictions and experimental measurements for the concentration of nicotine in the indoor ambient air 1 metre from the smoking machine when aerosols were generated from three different e-cigarettes (31 µg, 56 µg or 58 µg nicotine delivered per puff) with two variants of emission pattern (“low”, 7 puffs or “high”, 15 puffs) under restricted room ventilation (**B**) or intensive room ventilation (**C**). Distribution of model predictions is shown in grey range bar graphs and experimental measurements in white.

Using the experimental values and experimental design described above, the air quality model presented in this paper can be used to simulate the change in concentration of nicotine in the ambient air over time under the different experimental conditions (3 e-cigarette brands × 2 variants of emission pattern × 2 variants of room ventilation). In the above experiments, the released aerosol was machine generated therefore the retention rate of nicotine would be 0%, and 100% release of nicotine to the air. If one assumes the released aerosol fills the volume of the room rapidly, *i.e.*, in 1 min due to the air mixing by the fans, the released aerosol would propagate at 2 m/min.

In experiments using machine generated vapour [2], the average 1 h concentration of nicotine in the air was measured to range from 0.82 to 6.23 µg/m^3^ across the 12 scenarios, with little difference observed between restricted room ventilation (range from 0.88 to 6.23 µg/m^3^) and intensive room ventilation (range from 0.82 to 4.52 µg/m^3^), suggesting other parameters such as deposition also make a contribution [21]. By comparison, the air quality model predicted the average 1 h concentration of nicotine in the air to range from 0.75 to 6.29 µg/m^3^ across all the scenarios with an assumed of speed of nicotine deposition of 0.06 m/min. 

This deposition speed is in general agreement with that previously reported for pure nicotine vaporised in indoor air [22]. Figure 3A represents the model output for the two extreme experimental conditions (least extreme: low nicotine delivery + low emission pattern + intensive room ventilation; most extreme: high nicotine deliver + high emission pattern + restricted room ventilation). The model predicted the concentration of nicotine in the indoor air to range from 1.57 to 6.29 µg/m^3^ with restricted room ventilation (Figure 3B) and 0.75 to 3.01 µg/m^3^ with intensive room ventilation (Figure 3C). Taken together, this indicates there is overall good agreement between model predictions and experimental measurements and demonstrates the capacity of this modelling approach to reproduce the range of experimental observations.

### 3.4. An Illustrative Exposure Simulation: Cumulative Nicotine Exposure over an 8 h Working Day

**Figure 4:**
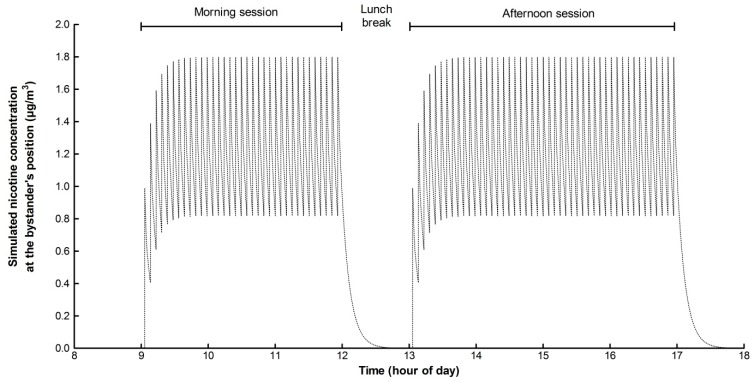
Cumulative effect of each single “puff” over an 8 h working day: calculation of bystander exposure to nicotine in ambient air. In this office scenario, it is assumed: an non e-cigarette user (the bystander) is seated 2 m from a colleague who “puffs” once every 5 min over an 8 h working day (9:00 h to 17:00 h with a one hour lunch break); the e-cigarette user inhales 30 µg nicotine per puff and exhales an aerosol which propagates in the indoor ambient air at 0.6 m/min; the exhaled aerosol deposits at a speed of 0.06 m/min and the room ventilation rate is 1.33 air changes per hour.

In order to illustrate the flexibility of the modelling approach, here we assessed the concentration of nicotine in the ambient air in a simulated office space during use of an e-cigarette to estimate bystander exposure to nicotine in exhaled e-cigarette aerosol over a working day (Figure 4).

In this scenario, two employees working in the same office were considered, spending 7 h per day together with a 1 h lunch break. The office size was 15 m^2^ and its volume 37.5 m^3^, equivalent to the size of a small office environment. The non-vaping worker (the bystander) was seated 2 m from a colleague who “puffed” once every 5 min of the course of the working day (the e-cigarette user). By way of context, this puffing frequency would lead to a consumer uptake of nicotine equivalent to a nicotine patch as reported previously [23]. E-cigarettes have been shown to deliver a range of nicotine from 2 µg to 56 µg per puff [20]; in order to provide an upper-bound bystander nicotine exposure estimation, we therefore assumed the e-cigarette user inhaled 60 µg of nicotine per puff. Although the quantity of nicotine retained by e-cigarette users remains to be determined experimentally, it is likely to be influenced by the e-cigarette consumers individual use pattern and behaviour (also termed “topography”). For example, if the user typically inhales (*i.e.*, inhaling deeply) or puffs the product (*i.e.*, taking vapour only into the mouth), frequency of puff, puff volume will all affect retention. It is unlikely the user retains 0% or 100% of the nicotine in the aerosol; some studies have reported 80% nicotine retention within the human body for a nicotine-containing aerosol [24]. However, due to the lack of published data on nicotine retention following e-cigarette use, and again to provide upper-bound estimates of bystander nicotine exposure, in this scenario it was assumed 50% of the nicotine was retained by the user per puff. A scenario of no-retention (*i.e.*, total re-exhalation into the room) can therefore be calculated by doubling the final value. Therefore, following intermittent use of the e-cigarette, the user will exhale 30 µg of nicotine per puff into the indoor ambient air. The exhaled aerosol will propagate in the ambient air due to convection movements leading to progressive dilution of the aerosol [25]. Experimentally, [25] showed through measurements that it could take up to 100 min for a 31 m^3^ space to be filled under isothermal conditions, but only 7 to 15 min with the additional effects of a heater in absence of forced ventilation. Under these conditions, the speed of propagation would have to be 0.27 m/min in all direction to fill the room in 10 min. Other studies [26] found that median values of the speed of indoor air were in the range 3.4 to 9 m/min. Such speeds are however multidirectional, and are not directly representative of the net speed of aerosol propagation in a room. It was assumed here that the ventilation producing the air exchange generated movement by convection and aerosol propagation at a speed of 0.6 m/min, whereby a single exhaled puff would fill the volume of the room in 3 min. When the office air extraction mechanisms begin, this leads to a reduction in the concentration of the constituents in the air. In this scenario, a typical indoor air exchange rate of 50 m^3^/h was selected (*i.e.*, 1.33 air changes per hour). The nicotine deposition velocity was assumed to be 0.06 m/min, calculated from other data [2]. The final simulation is represented on Figure 4, where the cumulative peaks of bystander exposure are observed, as well as the rapid decrease in airborne nicotine levels during the lunch break.

Given a typical range of breathing rate of 8 to 16 L/min [27,28,29], over the course of the working time, the bystander breathes approximately 3360 to 6720 L, equivalent to 4032 to 8064 g of air. In this scenario, the maximum concentration of nicotine the bystander will be exposed to over the working day is approximately 1.8 µg/m^3^. By way of context, in the U.K., the workplace exposure limit for nicotine is 500 µg/m^3^ for average exposure intensity over 8 h in the workplace [30]. In absolute terms, over the working day, the total quantity of nicotine potentially inhaled by the bystander would be approximately 4 to 8 µg. This is substantially, lower than the active consumption of the e-cigarette user, who in the same period has inhaled a total of 6108 µg nicotine, *i.e.*, 763 to 1527 times more. The model predictions suggest that under this scenario, the exposure of bystanders to nicotine in the exhaled aerosol is not at levels that would be expected to cause health concerns, consistent with views expressed elsewhere [31]. A similar approach could be adopted to assess exposure to other exhaled aerosol constituents and the corresponding quantity inhaled by the bystander. For example, it has been reported in the literature formaldehyde may be released from some e-cigarettes [32].

### 3.5. Effect of Varying All Model Parameters on the Concentration of Nicotine in Ambient Air

**Figure 5:**
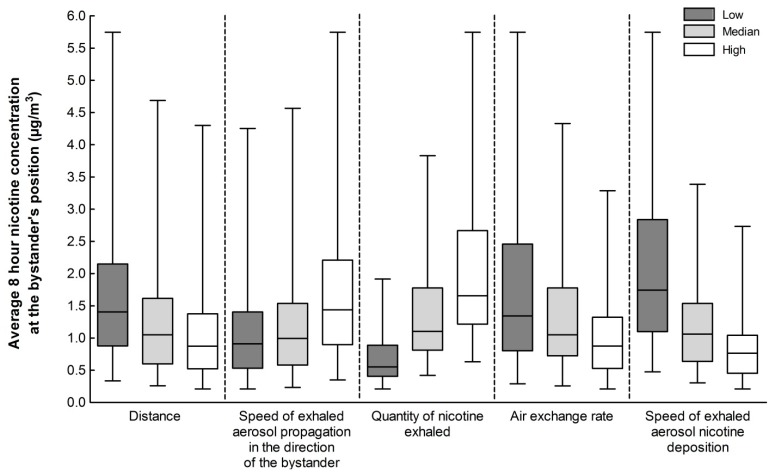
Impact that each parameter has on the calculated range of average 8 h nicotine concentrations in the ambient air at the bystander’s position. Each parameter is assigned three values: low (L), medium (M) or high (H). Distance of the bystander from the e-cigarette user, 1 (L), 1.5 (M) or 2 m (H); speed of exhaled aerosol propagation in the direction of the bystander, 0.5 (L), 1.0 (M) or 2.0 m/min (L); quantity of nicotine exhaled, 10 (L), 20 (M) or 30 μg (H); air exchange rate from 1 (L), 2 (M) or 3 (H) air changes per hour; and speed of exhaled aerosol nicotine deposition, 0.01 (L), 0.03 (M) and 0.06 m/min (H). Boxes represent the 25th and 75th percentiles, lines inside the boxes are medians and whiskers represent minimum and maximum values.

The impact of varying a single parameter in the model on the concentration of nicotine in the ambient air at the bystander’s position was evaluated above (Figure 2). Here we explore the model further by considering the collective effect of varying all parameters concurrently over the 8 h working day in the scenario described above. A dataset was generated by assigning each parameter within the model three values, “low (L)”, “medium (M)” and “high (H)”, resulting in a total of 243 unique modelled scenarios which collectively predicts the range of average nicotine concentrations in ambient air over 8 h in the workplace. Across all 243 scenarios, the lowest average 8 h nicotine concentration in the ambient air was 0.2 μg/m^3^ where quantity of nicotine exhaled, L; speed of exhaled aerosol propagation in the bystander’s direction, L; speed of exhaled aerosol nicotine deposition, H; distance from e-cigarette user, H; and indoor air exchange rate, H. Conversely, the highest average 8 h nicotine concentration in the ambient air was 5.8 μg/m^3^, where quantity of nicotine exhaled, H; speed of exhaled aerosol propagation in the bystander’s position, H; speed of exhaled aerosol nicotine deposition, L; distance from e-cigarette user, L; and indoor air exchange rate, L. Next we considered the impact that each parameter has on the calculated range of average 8 h nicotine concentrations. To evaluate the effect of parameter A, the 243 scenarios were grouped where parameter A was constant *i.e.*, low (81 scenarios), medium (81 scenarios) and high (81 scenarios). Changes in the maximum average 8 h nicotine concentration between the “low” and “high” parameter values were then used to assess the impact that parameter A has on the calculated 8 h average nicotine concentration ranges. As shown in Figure 5, all model parameters had an impact on the maximum 8 h average nicotine concentration in the ambient air. 

An increase in the “quantity of nicotine exhaled” or “speed of exhaled aerosol propagation in the direction of the bystander” resulted in an increase of 67% or 26% in the maximum average 8 h nicotine concentration in ambient air at the bystander, respectively. An increase in the “speed of exhaled aerosol deposition”, “indoor air exchange rate” or “distance from e-cigarette user” resulted in a 52%, 43% or 25% reduction in the maximum average 8 h nicotine concentration in ambient air at the bystander position, respectively. In all cases, the maximum average 8 h nicotine concentration in ambient air at the bystander was significantly lower than the UK workplace exposure limit for nicotine [30]. The most important model parameter identified with regard to bystander exposure was found to be the “quantity of nicotine exhaled”. Therefore, it is essential that precise measurements are made regarding the quantity of nicotine retained by the e-cigarette user, *i.e.*, the fraction not exhaled into the ambient air, when determining bystander exposure to nicotine in exhaled e-cigarette aerosol. 

## 4. Conclusions

The increasing use of e-cigarettes necessitates an understanding of how these and similar products affect indoor air quality [5,6]. While a number of studies have investigated e-cigarettes in general, there are few studies that have reported on indoor air quality and potential bystander effects [3,4,33].

Conducting experimental studies are costly and time consuming and it can be challenging to extrapolate or generalise to other scenarios. Here, a simple indoor air quality model is reported that explores the issue in a fundamental way by applying mass-balance approaches. The result of this is a model which describes the concentration of exhaled e-cigarette aerosol constituents in ambient air over time, from which bystander exposure to that constituent can be estimated. It has been reported recently that use of an e-cigarette increases exposure of non-smokers and bystanders to nicotine and a number of other toxicants in the ambient air which may pose a health risk [7]. In this paper, we explored bystander exposure to nicotine; however the model can be readily applied to other constituents of interest which may be present in the exhaled aerosol, for example formaldehyde.

The model appears to perform well when compared to published experimental values for nicotine in the indoor ambient air following generation and release of an e-cigarette aerosol. Under both the modelled and experimental scenarios, the levels of nicotine in the ambient air are substantially lower than workplace exposure limits and would not be anticipated to pose a health concern, a finding in agreement with [31]. However, additional refinement is necessary to enhance the accuracy of the model outputs and to enable its use as a predictive tool; it is a central maxim of model development that the quality of the output is predicated on the quality of the inputs.

For example, while it is possible to consider zero retention by the e-cigarette user, *i.e.*, 100% exhalation of the constituent, to derive maximal values it would be preferable to understand the typical range, since this would also inform on potential surface deposition of the constituent. In the case of nicotine, it has been reported recently that following generation of an e-cigarette aerosol using a machine smoking protocol, *i.e.*, resulting in 0% nicotine retention and 100% release of nicotine to the air, nicotine is deposited on various surfaces [21]. Underscoring the need for this refinement was the finding in our assessment of the contribution from the different parameters, which showed that the quantity of nicotine exhaled was most important influencing bystander exposure. As listed in Table 2, additional refinements may also include understanding typical user behaviours to ensure that exposures from real-life vaping patterns are considered.

The simple air quality model presented here may be used to investigate the concentration of pollutants in indoor ambient air following use of an e-cigarette given emission data for an e-cigarette and its user and the characteristics of the room. This capacity can be a useful approach for preliminary, cost-effective evaluation of the potential indoor air pollution impacts with use of an e-cigarette and thus estimating a bystander’s potential exposure.

**Table 2:** Needs identified for further experimental studies.

Phase	Topic
Inhalation/Exhalation	Characterisation of e-cigarette user vaping behaviour: puff volume, frequency and inhalation (“topography”). Dynamic of the aerosol in the air pathways: condensation, evaporation, absorption and release. Characterisation of the ranges of retention rates for the various aerosol constituents.
Aerosol propagation/dilution	Dynamic of the aerosol in the air during propagation: condensation, evaporation and gravimetric effects Determination of the typical speeds of propagation under air change regulatory conditions
Bystander exposure	Assessment of the exposure level of concern for the various aerosol constituents. Evaluation of the impact of the distance between user and non-user.
Air exchange	Characterisation of the absorption and desorption dynamics *versus* environmental conditions, *i.e.*, surface materials, air speed, air exchange. Investigations of the possible surface chemical reactions.
Bystander exposure dose	Study of the typical scenario of exposure in different locations *i.e.*, work, home or restaurant. Assessment of the dose levels of concern for the chemical.

## Supplementary Files

Supplementary File 1
