# Supplementary material for: Electronic Cigarettes and Indoor Air Quality: A Simple Approach to Modeling Potential Bystander Exposures to Nicotine
Source: Int J Environ Res Public Health. 2014 Dec 24;12(1):282–99. doi: 10.3390/ijerph120100282 (PMC4306862; doi:10.3390/ijerph120100282)
Supplement: Supplementary File 1 [file ijerph-12-00282-s001.pdf]

Electronic Cigarettes and Indoor Air Quality: A Simple Approach to Modeling Potential Bystander Exposures to Nicotine

Mathematical Formulation

First, the e-cigarette user inhales a certain quantity of aerosol constituent and exhales a different quantity depending on the retention rate in the air pathways as expressed with Equation (1):

$$M_{Constituent\_Exhaled} = M_{Constituent\_Inhaled} \times \left(1 - Ret_{\%}\right) \tag{1}$$

where $M_{Constituent \ \ Inhaled}$ = the quantity of constituent inhaled (µg),

$Ret_{\%}$ = the retention rate by the body (%), and

$M_{Constituent \ \ Inhaled}$ = the quantity of constituent exhaled (µg).

The concentration of constituents in the volume exhaled is obtained from the ratio of the quantity to the volume exhaled as expressed by Equation (2). The initial time $t = 0$ is defined as the time of the puff exhalation:

$$C_{Constituent}\left(t = 0\right) = \frac{M_{Constituent\_Exhaled}}{V_{Exhaled}} \tag{2}$$

where $V_{Exhaled}$ = the volume exhaled (m^3)

$C_{Constituent}$ = the concentration of aerosol constituent in volume filled (µg/m^3)

The shape of the exhaled aerosol is arbitrarily assumed to fill a cube at t = 0. This assumption made for simplicity purposes is justified by the fact that no shape is better than another; none represents perfectly the real shape. In fact, regarding exposure, the main key factor is the speed at which the aerosol will propagate in space, not the initial shape. Each dimension of the cube increases at given speeds due to multi-directional convection movements, and it is considered that the aerosol will fill homogeneously a rectangular cuboid progressively expanding. The change of the volume of aerosol over time $V_{Aerosol}(t)$ within the limits of the room can be calculated from the speeds of propagation in all directions.

Spatial referential is illustrated in Figure S1. Positions in space are defined by the coordinates x, y and z along the three Cartesian dimensions. The origin of the referential is defined at the deep lower corner of the room. The signs −/+ are used to indicate the sense of propagation.

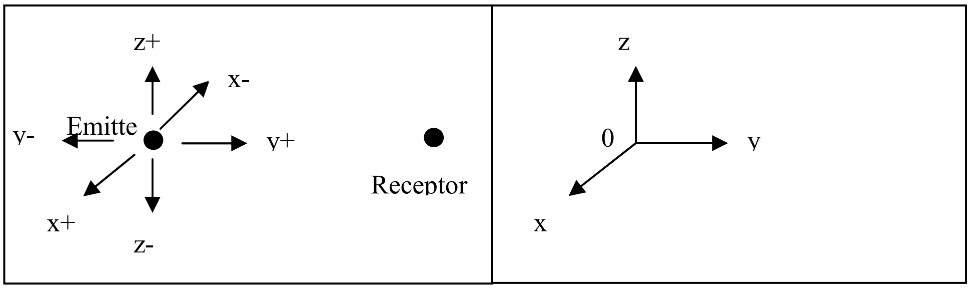

Figure S1. Referential and coordinates in the room space.

The dimension of the cube just after exhalation can be obtained with the following expression:

$$d_{x-}(0) = d_{x+}(0) = d_{y-}(0) = d_{y+}(0) = d_{z-}(0) = d_{z+}(0) = \frac{\sqrt[3]{V_{Exhaled}(t=0)}}{2} \quad (3)$$

The parameters $d_{x-}(0)$... represent the distance of the faces of the cube to the emitting point. The coordinates of the emitter(s) are x_{Em}; y_{Em}; z_{Em}. The coordinates of the receptor(s) are x_{Rec}; y_{Rec}; z_{Rec}. The limits of the room are given by x_{max}; y_{max}; z_{max}. Taking into account the speeds of propagation $Sp_{x-}(t)$, …, in all dimensions, and the physical limits of the room, the change of the volume of aerosol over time is given by:

$$V_{Aerosol}(t) = [d_{x-}(t) + d_{x+}(t)] \times [d_{y-}(t) + d_{y+}(t)] \times [d_{z-}(t) + d_{z+}(t)] \quad (4)$$

with the following conditions:

If $Sp_{x-} > 0$ then $d_{x-}(t) = \min\left[d_{x-}(0) + \int_0^t Sp_{x-}(t).dt; x_{Em} \right]$

$$d_{x-}(t) = \min\left[d_{x-}(0) + \int_0^t Sp_{x-}(t).dt; x_{\max} - x_{Em} \right] \text{ else} \quad (5)$$

If $Sp_{x+} > 0$ then $d_{x+}(t) = \min\left[d_{x+}(0) + \int_0^t Sp_{x+}(t).dt; x_{\max} - x_{Em} \right]$

else $d_{x+}(t) = \min\left[d_{x+}(0) + \int_0^t Sp_{x+}(t).dt; x_{Em} \right]$

Similar expressions apply to y and z. The speeds $Sp_{x-}(t)$… should be derived from experiments. A random component, with a zero average, could be added in order to simulate realistic variations of air propagation in a natural environment. This would add variance but would not change the calculations on average. The change in concentration of the aerosol constituents during the phase of propagation is simply the ratio of the quantity exhaled to the volume progressively filled, as expressed with Equation (6):

$$C_{Constituent}(t) = \frac{M_{Constituent\_Exhaled}}{V_{Aerosol}(t)} \quad (6)$$

Once the exhaled constituent has filled a certain proportion of the room, a quantity starts to be extracted due to ventilation. The airflow of extraction is derived from the air exchange rate ACH and the volume of the room. If the air extracted is partly recycled, a recycling factor RRA needs to be included in the calculation. The absolute airflow of extraction is given by Equation (7):

$$Q_{Extract} = \frac{ACH \times V_{Room}}{60} \times (100 - RRA) \quad (7)$$

where V_{Room} = the volume of the room (m^3),
ACH = the air exchange rate (/h),
$Q_{Extract}$ = the extraction airflow (m^3/min), and
RRA = the recycling rate of extracted air (%).

The quantity extracted over time can be calculated from the following equation:

$$M_{Extract}(t) = \int_0^t Q_{Extract} \times C_{Constituent}(t).dt \tag{8}$$

where $M_{Extract}$ = the quantity extracted (µg).

Extraction is not the only factor reducing the quantity of aerosol constituents in air; surface deposition can also contribute to reduction. The deposition velocity v_d is the net flux density of a constituent to a surface divided by the concentration in the air. This velocity depends on a number of factors like aerosol particle size, surface natures or environmental conditions, and has been successfully simulated mathematically [1]. From a macroscopic point of view, the equivalent airflow of deposition is the multiplication of the surface by the speed as expressed by Equation (9):

$$Q_{Dep} = S_{Dep} \times v_d \tag{9}$$

where Q_{Dep} = the net deposition flow (m^3/min),
S_{Dep} = the surface of deposition (m^2), and
v_d = the deposition velocity (m/min).
S_{Dep} is at least equal to the surface of the walls:

$$S_{Dep} \geq 2 \times [(x_{\max} \times y_{\max}) + (x_{\max} \times z_{\max}) + (y_{\max} \times z_{\max})] \tag{10}$$

The quantity deposited on the walls over time can be calculated from the following equation:

$$M_{Dep}(t) = \int_0^t Q_{Dep} \times C_{Constituent}(t).dt \tag{11}$$

where M_{Dep} = the deposited quantity (µg).

The surface concentration can be easily derived from M_{Dep}. When both air extraction and surface deposition occur, the change of aerosol constituent in the ambient air can be expressed with the differential Equation (12):

$$\frac{dM_{Constituent}(t)}{dt} = -Q_{Extract} \times C_{Constituent}(t) - Q_{Dep} \times C_{Constituent}(t) \tag{12}$$

where $M_{Constituent}$ = the remaining quantity of aerosol constituent exhaled (µg).

The volume of the room being constant, the change over time of aerosol constituent concentration is proportional to the change of the remaining quantity:

$$\frac{dM_{Constituent}(t)}{dt} = V_{Room} \times \frac{dC_{Constituent}(t)}{dt} \tag{13}$$

From Equations (12) and (13), the differential Equation (14) can be derived:

$$V_{Room} \times \frac{dC_{Constituent}(t)}{dt} = -(Q_{Extract} + Q_{Dep}) \times C_{Room}(t) \tag{14}$$

The solution of Equation (14) is given by Equation (15):

$$C_{Constituent}(t) = C_{Constituent\_Init} \times e^{-[a \times (t - t_{Init})]} \tag{15}$$

where $a = \frac{Q_{Extract} + Q_{Dep}}{V_{Room}}$

$C_{Constituent\_Init}$ = the constituent concentration when extraction and/or deposition starts (µg/m^3), and

t_{Init} = the time at which the extraction/deposition effects start (min).

The air convection movements accelerate the propagation at a first stage and the extraction/deposition reduce further the indoor air concentration at a later stage, *i.e.*, only once the constituent fills a certain percentage p_{Room} of the whole volume at the time t_{Init}. Both simultaneous effects of dilution and extraction during a certain period of time can be merged into a single expression as given with Equation (16):

$$C_{Constituent}(t) = C_{Constituent\_Init} \times \frac{V_{Aerosol}(t_{Init})}{V_{Aerosol}(t)} \times e^{-[a \times (t - t_{Init})]} \quad (16)$$

In reality, the effects of extraction and deposition do not necessarily start simultaneously. Once the exhaled constituent has filled a sufficient proportion of the room at a time $t_{Extract\ Init}$, a certain quantity starts to be extracted by the ventilation system. Similarly to the extraction case, once a certain proportion is filled at a time $t_{Dep\ Init}$, a certain quantity is likely to be deposed on the surfaces. The coefficients "*a*" of the equation can then change over time.

For example, if extraction occurs before deposition $t_{Extract\_Init} < t_{Dep\_Init}$, $a = \frac{Q_{Extract}}{V_{Room}}$ from $t = t_{Extract\_Init}$ and then $a = \frac{Q_{Extract} + Q_{Dep}}{V_{Room}}$ from $t = t_{Dep\_Init}$.

The temporary effect of a door or window opening can be assessed by considering a coefficient "*a*" changing over time *a(t)*. This coefficient becomes higher when a door or a window is open because additional air goes in, and aerosol goes out. A convenient expression of the concentration change over time is given by:

$$C_{Constituent}(t_i) = C_{Constituent}(t_{i-1}) \times \frac{V_{Aerosol}(t_0)}{V_{Aerosol}(t_i)} \times e^{-[a(t_i) \times (t_i - t_{i-1})]} \quad (17)$$

where $a(t_i)$ levels follow the series of events

At the position of the e-cigarette user, the concentration after exhalation is first impacted by dilution, and then by air exchange and surface deposition. This can be translated into Equations as follow :

If $V_{Aerosol}(t) < p_{Room} \times V_{Room}, C_{Constituent\_User}(t) = \frac{M_{Constituent\_Exhaled}}{V_{Aerosol}(t)}$

when $V_{Aerosol}(t) = p_{Room} \times V_{Room}$ at $t = t_{Init}$,

$$C_{Constituent\_User}(t) = C_{Constituent\_User}(t_{Init}) \times \frac{V_{Aerosol}(t_{Init})}{V_{Aerosol}(t)} \times e^{-[a \times (t - t_{Init})]} \quad (18)$$

where $a = \frac{Q_{Extract} + Q_{Dep}}{V_{Room}}$.

For the bystander, the exposure does not start before the aerosol constituent reaches his position. Several conditions have to be tested and can be summarized as follows:

$$\begin{aligned} x_{Em} - d_{x-}(t) \le x_{Rec} \le x_{Em} + d_{x+}(t) \\ y_{Em} - d_{y-}(t) \le y_{Rec} \le y_{Em} + d_{y+}(t) \\ z_{Em} - d_{z-}(t) \le z_{Rec} \le z_{Em} + d_{z+}(t) \end{aligned} \quad (19)$$

If the previous conditions are not satisfied, the exposure had not occurred start yet: $C_{Constituent\_Bys\tan der}(t) = 0$

If the previous conditions are satisfied and if $V_{Aerosol}(t) < p_{Room} \times V_{Room}$, *i.e.*, the aerosol had not filled the room sufficiently to be extracted, then:

$$C_{Constituent\_Bys\tan der}(t) = \frac{M_{Constituent\_Exhaled}}{V_{Aerosol}(t)} \quad (20)$$

Finally, if the previous conditions are satisfied, and if $V_{Aerosol}(t) \geq p_{Room} \times V_{Room}$ then:

$$C_{Constituent\_Bys\tan der}(t) = C_{Constituent\_Bys\tan der}(t_{Init}) \times \frac{V_{Aerosol}(t_{Init})}{V_{Aerosol}(t)} \times e^{-[a \times (t - t_{Init})]} \quad (21)$$

where t_{Init} corresponds to. $V_{Aerosol}(t_{init}) = p_{Room} \times V_{Room}$

Exposure at the between a time t_1 and a time t_2 is defined by the sum of the surrounding aerosol concentration:

$$E = \int_{t1}^{t2} C_{Constituent}(t).dt \quad (22)$$

The total quantity inhaled by the e-cigarette user is given by Equation (23) :

$$M_{Tot\_Inhaled\_User} = \sum_{1}^{N_{Puff}} M_{Constituent\_Inhaled} + \int_{0}^{t} C_{Constituent\_User}(t) \times V_{Inhaled}.dt \quad (23)$$

The total quantity inhaled by the bystander is given by Equation (24) :

$$M_{Tot\_Inhaled\_Bystander} = \int_{0}^{t} C_{Constituent\_Bystander}(t) \times V_{Inhaled}.dt \quad (24)$$

Reference

1. Nazaroff, W.W.; Cass, G.R. Mathematical modelling of indoor aerosol dynamics. *Environ. Sci. Technol.* **1989**, *23*, 157–166.

© 2014 by the authors; licensee MDPI, Basel, Switzerland. This article is an open access article distributed under the terms and conditions of the Creative Commons Attribution license (http://creativecommons.org/licenses/by/4.0/).
